# Introduction of 3D-classification and its derived surgical sequence of Schatzker type IV tibial plateau fractures

**DOI:** 10.1186/s12893-023-02284-0

**Published:** 2023-12-09

**Authors:** Zihao Liu, Yanlong Zhang, Shengjie Wang, Shuai Wang, AQin Peng

**Affiliations:** 1 Department of Orthopaedic Trauma, Harrison International Peace Hospital, Hengshui, 053000 Hebei China; 2 Department of Orthopaedic Surgery, Third Hospital of Hebei Medical University, No. 139 Ziqiang Road, Shijiazhuang, 050051 Hebei China; 3 Department of Orthopaedic Surgery, Hebei Chest Hospital, NO.372 Shengli Road, Shijiazhuang, 050051 Hebei China; 4 Department of Trauma Surgery, First Hospital of Hebei Medical University, Shijiazhuang, 050051 Hebei China

**Keywords:** Classification, Schatzker type IV tibial plateau fractures, Surgical sequence

## Abstract

**Introduction:**

Schatzker IV tibial plateau fractures usually have a worse prognosis due to their high variability and the accompanied bony and soft tissue injuries. This study aimed to introduce an injury mechanism-based new classification of Schatzker IV tibial plateau fractures and evaluate its reliability. Additionally, this study aimed to evaluate the outcomes of operative Schatzker IV tibial plateau fractures treated according to the surgical sequences determined by the new classification.

**Materials and methods:**

A total of 63 cases of operative Schatzker IV tibial plateau fractures that were treated following the new surgical sequences were enrolled in our study. The CT images of these patients were reviewed and classified twice according to the new 3D classification by 4 independent observers. The reliability of the classification was calculated through kappa analysis. The classification-determined surgical sequence was evaluated by observing the postoperative efficacy during the follow-up.

**Results:**

Both the intra-observer (the mean k = 0.897, CI 0.806–0.971) and inter-observer (the mean k = 0.883, CI 0.786–0.961) reliability of 3D-classification showed excellent agreement according to Landis and Koch. All the patients were followed up for 6–28 months (average 12.8 months). As for the evaluation of the postoperative efficacy, according to KSS, 53 cases were rated as excellent, 8 cases as good, and 2 cases as fair results.

**Conclusions:**

The new proposed classification showed high intra-observer and inter-observer reliability in our study. The surgical sequence determined by the classification can help surgeons to acquire good reduction and rigid internal fixation. Therefore the new classification of Schatzker IV tibial plateau fractures and the derived surgical sequences are worthy of further popularization and application in clinical trials.

## Introduction

Tibial plateau fractures, belonging to intra-articular injuries, present complex clinical features, rendering their treatments challenging for orthopedic surgeons [1, 2]. In terms of tibial plateau fracture treatment, reducing and retaining multiple articular fragments, restoring axial alignment, anatomically reconstructing the joint surface, and achieving knee stability without compromising soft tissues are still challenging [3].

Currently, there are at least 38 popularized classifications worldwide for thoroughly interpreting the injury mechanism [4]. Among thses classifications, the Schatzker classification system is the most commonly used, which classifies tibial plateau fractures into six categories [5]. In the Schatzker classification system, Roman numerals represent the severity of injury. However, type IV fracture are later considered to indicate the worst prognosis, which can be attributed to their high variability, accompanied by bony and soft tissue injuries [6]. Schatzker IV tibial plateau fractures account for approximately 10% of all tibial plateau fractures [7]. Schatzker type IV fracture has been subtyped by accumulating orthopedics experts. For example, Wahlquist et al. have classified medial tibial plateau fractures into 3 types: A, B, and C [8]. According to the Three-Column Classification proposed by Luo, Schatzker type IV fractures can be classified as “two-column fractures”, involving the medial column and posterior column [9]. Moore has classified Schatzker type IV tibial plateau fractures into Moore type I or Moore type II [10]. However, few has been reported about the injury mechanism associated with the classification. Our team member, Wang [11], has explored the mechanism of Schatzker IV tibial plateau fractures using CT-based 3D models; as a result, 8 possible injury patterns were summarized, including hyperextension-varus-internal rotation, hyperextension-valgus-external rotation, extension-varus, extension-valgus, flexion-varus-internal rotation, flexion-varus-external rotation, flexion-valgus-internal rotation, and flexion-valgus-external rotation.

In this study, the injury patterns were defined as a new classification, 3D classification. Wang has described the 3D models-based injury patterns using Mimics software [11]. Moreover, the 3D classification was intelligible with the CT features of each injury pattern. The identified key points mainly include the main fracture apex (MFA), the main compression zone (MCZ), and the main orientation of fracture line (MOFL). These points can not only help orthopedists to sub-classify Schatzker type IV tibial plateau fractures but also influence the surgical sequences during the operation. This study introduced the new 3D classification of Schatzker IV tibial plateau fractures and evaluated its reliability. Additionally, this study aimed to evaluate the outcomes of operative Schatzker IV tibial plateau fractures that were treated with the surgical sequences determined by our new 3D classification.

## Materials and methods

### Patients

This retrospective study was carried out at our Academic Trauma Center from January 2018 to October 2020 and was approved by the Institutional Review Committee of the Ethics Committee. A total of 89 patients with Schatzker type IV tibial plateau fractures treated according to the new surgical sequences were retrospectively reviewed. All participants provided the informed consent.

The exclusion criteria were: [1] ipsilateral periarticular fracture (i.e., distal femoral fracture, femoral condylar fracture, and patellar fracture); [2] open or pathological knee fractures; [3] fractures in skeletally immature patients; [4] a history of knee surgery; [5] incomplete image data; and [6] loss of follow-up. After excluding the substandard cases, a total of 63 patients were finally enrolled.

### Description of 3D-classification

According to the 3D classification analysis conducted by Wang [11], Schatzker type IV tibial plateau fractures can be classified into 8 types: hyperextension-varus-internal rotation, hyperextension-valgus-external rotation, extension-varus, extension-valgus, flexion-varus-internal rotation, flexion-varus-external rotation, flexion-valgus-internal rotation, and flexion-valgus-external rotation. This classification relies on the identification of 3 key points: MFA, MCZ, and MOFL.

**Fig. 1:**
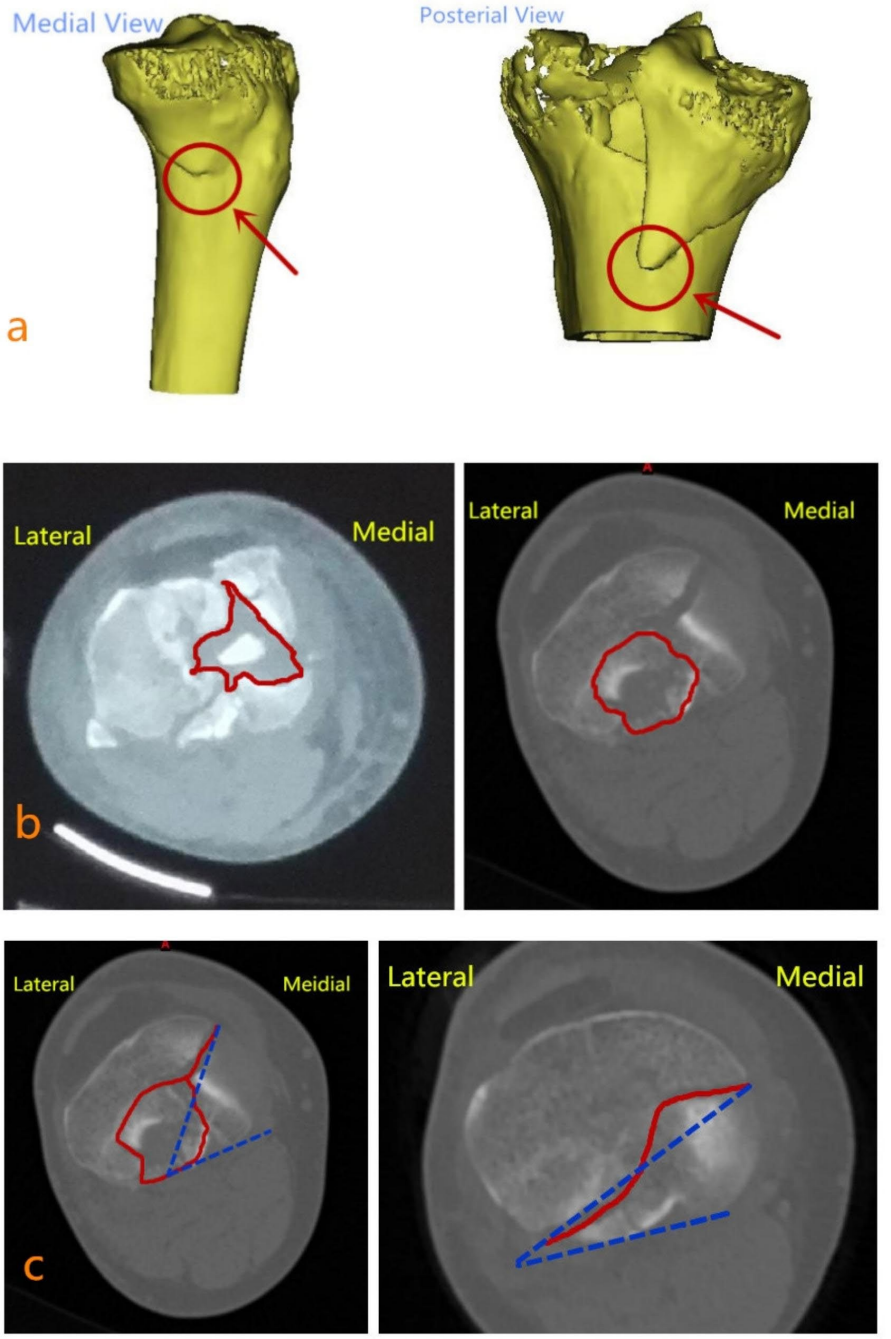
Three major identified points of IM Classification (MFA, MCZ, and MOFL). (**a**) The MFA of “extension” and “flexion” is located in the medial and posteromedial border, respectively. (**b**) The MCZ of “varus” and “valgus” is located in the medial and lateral tibial plateau, respectively. (**c**) The acute angle between MOFL of “internal rotation” and the posterior tibial condylar axis is greater than 50°, while that between MOFL of “external rotation” and the posterior tibial condylar axis is less than 45°

The first part of the classification (including “hyperextension”, “extension”, and “flexion”) is primarily determined by the MFA and MCZ. The MFA, located at the apex of the inverted triangle fragment, can be used to assess whether the fracture is an “extension” or a “flexion” type. Specifically, the MFA of the “extension” type is located in the medial border, while that of the “flexion” type is located in the posteromedial border. In cases of hyperextension-XX-XX, where an MFA is not present, the location of the MCZ is used to identify “hyperextension”. In short, the fracture can be classified as “hyperextension” if the MCZ is in the anteromedial quadrant or anterolateral quadrant. This MCZ feature did not apply to the other types (Fig. 1).

Furthermore, the MCZ can also be used to assess whether the fracture is “valgus” or “varus,“ which constitutes the second part of the classification. The fracture is “varus” if the MCZ is located in the medial tibial plateau or is not found; the fracture is “valgus” if the MCZ crosses the midline of the tibial plateau (Fig. 1).

The third part of the classification (“internal rotation” or “external rotation”) can be evaluated by the MOFL. In short, an acute angle between the MOFL and the posterior tibial condylar axis less than 45° indicated “external rotation,“ while that greater than 50° indicated “internal rotation” (Fig. 1).

**Fig. 2:**
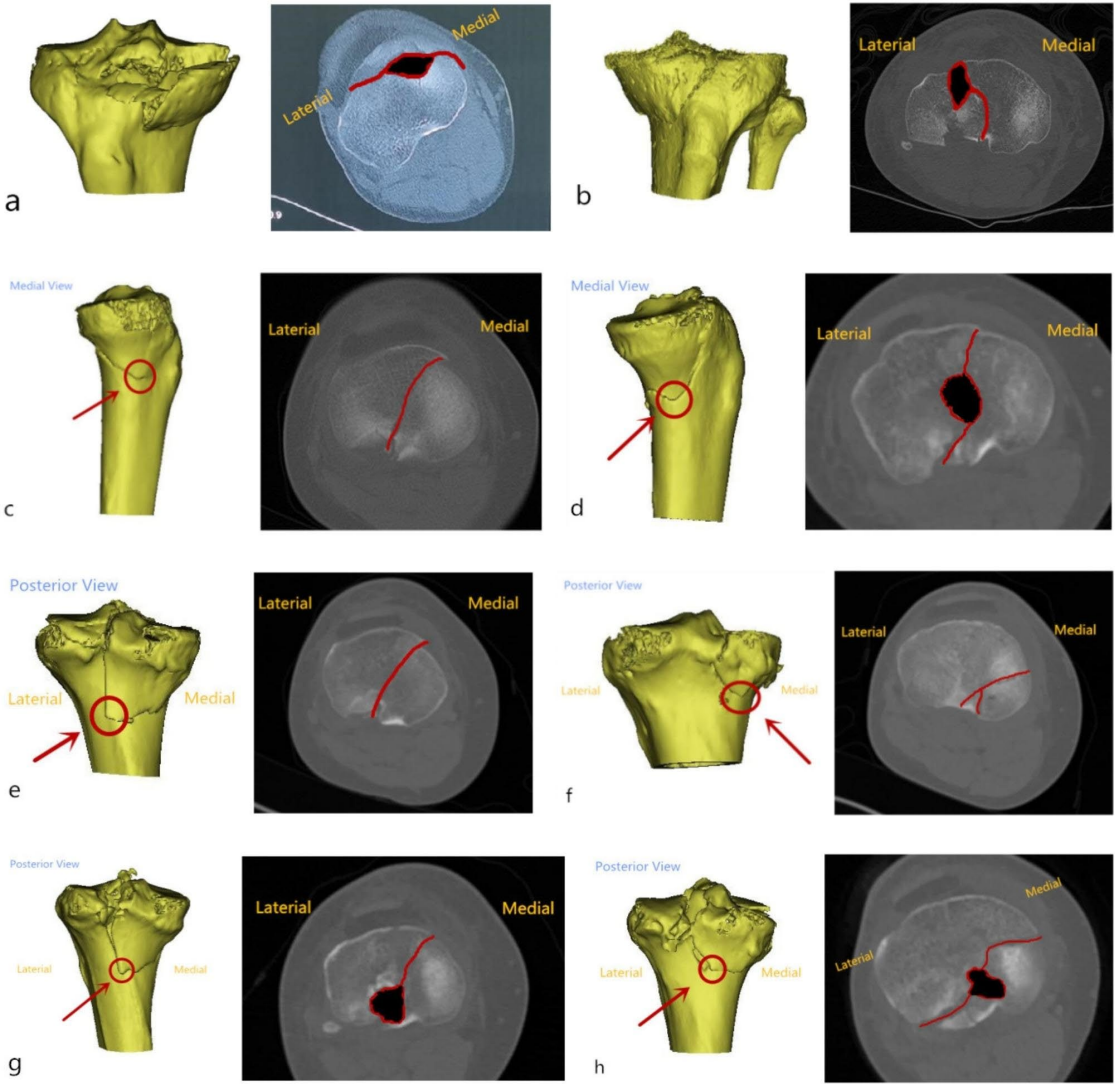
The main morphology of these 8 types was shown in CT images; the red arrows point to the MFA in 3D CT Reconstruction images, the red lines represent MOFL, and the black zones represent MCZ. (**a**) hyperextension-varus-internal rotation. (**b**) hyperextension-valgus-external rotation. (**c**) extension-varus. (**d**) extension-valgus. (**e**) flexion-varus-internal rotation. (**f**) flexion-varus-external rotation. (**g**) flexion-valgus-internal rotation. (h) flexion-valgus- external rotation

The CT features of the classification are summarized in Table 1. The main morphology of each type is displayed in Fig. 2.

**Table 1:** CT features of 3D Classification

Classification	n	Morphology	MFA	MCZ	MOFL
hyperextension-varus-internal rotation	4	Total/subtotal medial condyle fracture; sometimes combined with loss of posterior slope of the tibial plateau and avulsion fracture of fibular head;	Not found	Anteromedial	From anteromedia to medial
hyperextension-valgus-external rotation	1	Total medial condyle fracture;	Not found	Anterolateral	From anterolateral to posteromedial
extension-varus	9	Total medial condyle fracture;	Medial	Medial or not found	From anterior to posterior
extension-valgus	5	Total medial condyle fracture;	Medial	Lateral	From anterior to posterior
flexion-varus-internal rotation	6	Total/subtotal medial condyle fracture;	Posteromedial	Posteromedial or not found	From anteromedial to posteromedial
flexion-varus-external rotation	3	Subtotal medial condyle fracture;	Posteromedial	Posteromedial or not found	From medial to posteromedial
flexion-valgus-internal rotation	20	Total medial condyle fracture;	Posteromedial	Posterolateral	From anteromedial to posterolateral
flexion-valgus-external rotation	15	Subtotal medial condyle fracture;	Posteromedial	Posterolateral	From posteromedial to posterolateral

### Assessment of the reliability of the classification

The reliability of the classification was assessed by 4 independent observers (a senior orthopedist, a musculoskeletal radiologist, an attending doctor, and a resident). None of them were allowed to participate in the treatment and follow-up of patients. The observers received a detailed introduction of the 3D classification, ensuring that they fully understood and mastered the classification system. After that, the cases were randomly assigned to each observer for assessment with no feedback provided during the process. After 8 weeks, the assessment was repeated to ensure that each observer’s initial choice did not influence their subsequent evaluation. To maintain the data integrity, all the assessment results were collected and recorded by physicians who were not involved in the treatment and observation of patients.

### Use of the classification and its derived surgical sequence

The 3D classification can determine the surgical sequences to some extent in treating Schatzker type IV tibial plateau fractures.

#### Hyperextension-varus-internal rotation; hyperextension-valgus-external rotation

**Fig. 3:**
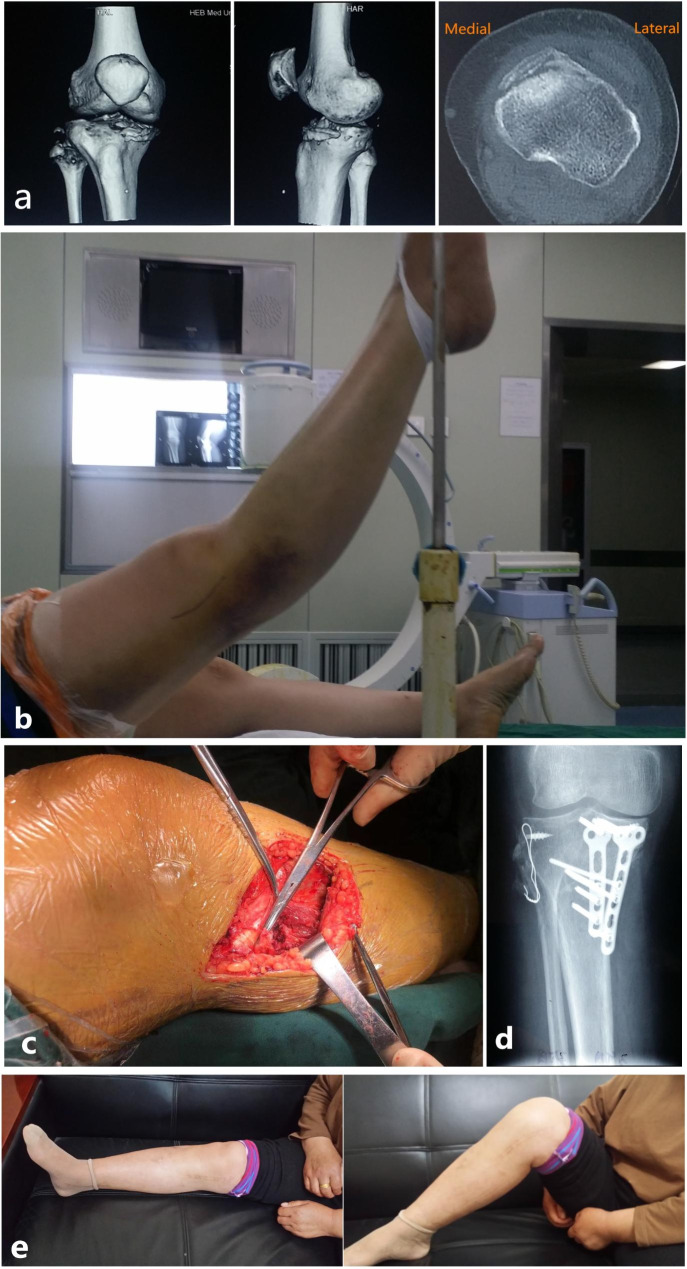
A typical case of a 42-year-old female with Schatzker IV tibial plateau fractures (hyperextension-varus-internal rotation type). (**a**) The features of this type were shown in CT images. (**b**) Preoperative hyperextension deformity suggested posterolateral instability of the knee joint. (**c**) The knee should be placed in flexion during the operation. (**d**) After restoring the compressed articular surface, two plates were needed for internal fixation. The avulsion fracture of the fibular head was fixed by rivets or wires. (**e**) 1 year postoperatively, the stability and function of the knee were good

Each patient was required in a supine position with the knee at about 45° of flexion. Considering the location of MCZ (in the anteromedial or anterolateral quadrant plateau), the anteromedial or anterolateral approach was used. A distrator was needed to restore to the normal posterior slope. Next, autogenous iliac bone graft and plate fixation were performed. Additionally, for patients with an avulsion fracture of fibular head or lateral collateral ligament injuries, rivets or wires were needed for the fixation of avulsion fracture and served ligaments could be sutured directly (Fig. 3).

#### Extension-varus; extension-valgus

**Fig. 4:**
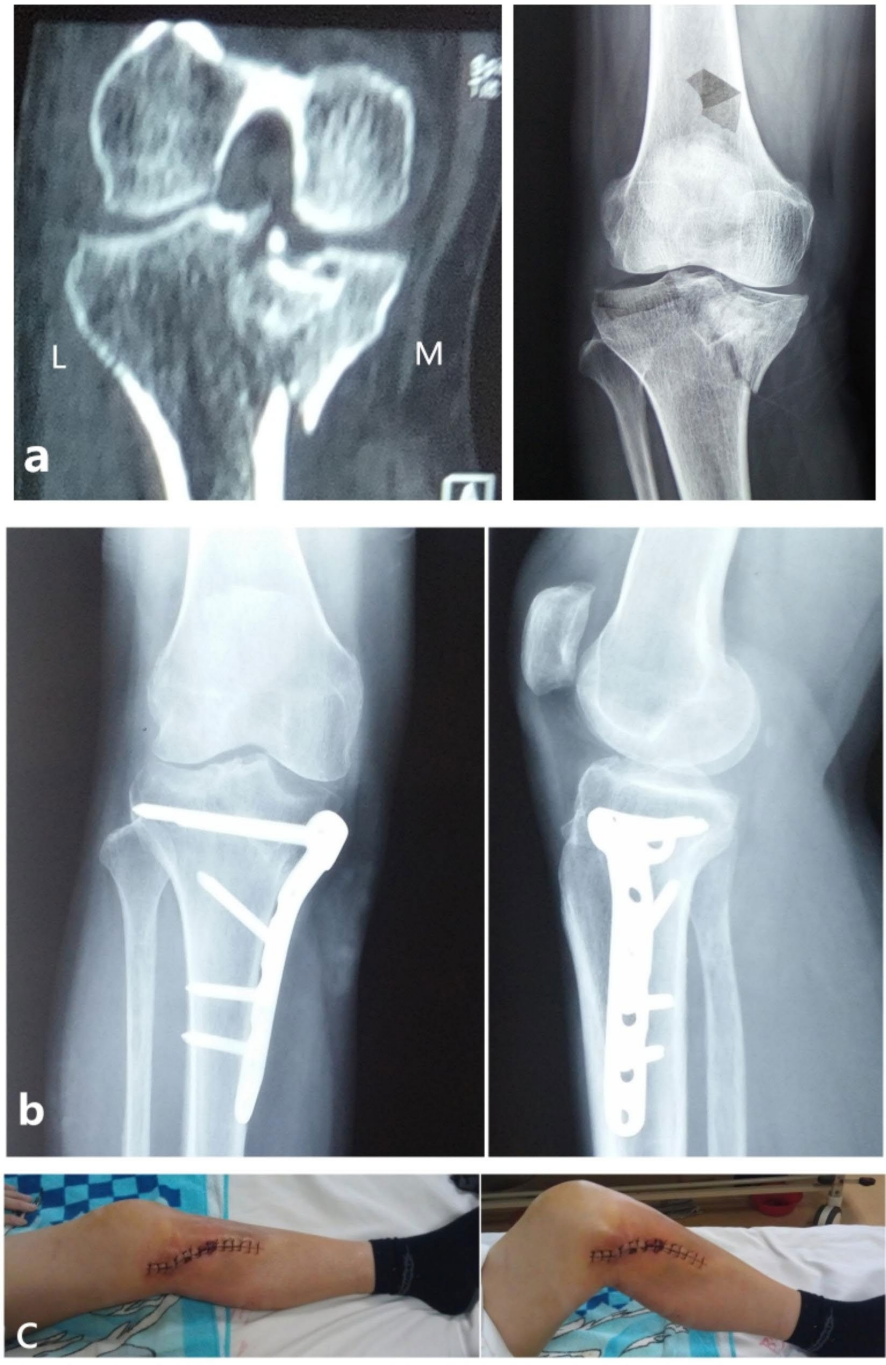
A typical case of a 45-year-old male with Schatzker IV tibial plateau fractures (extension-varus type). (**a**) The features of this type were shown in CT images. (**b**) After the reduction of MFA, a plate was needed for internal fixation. (**c**) Postoperatively, the function of the knee was good

**Fig. 5:**
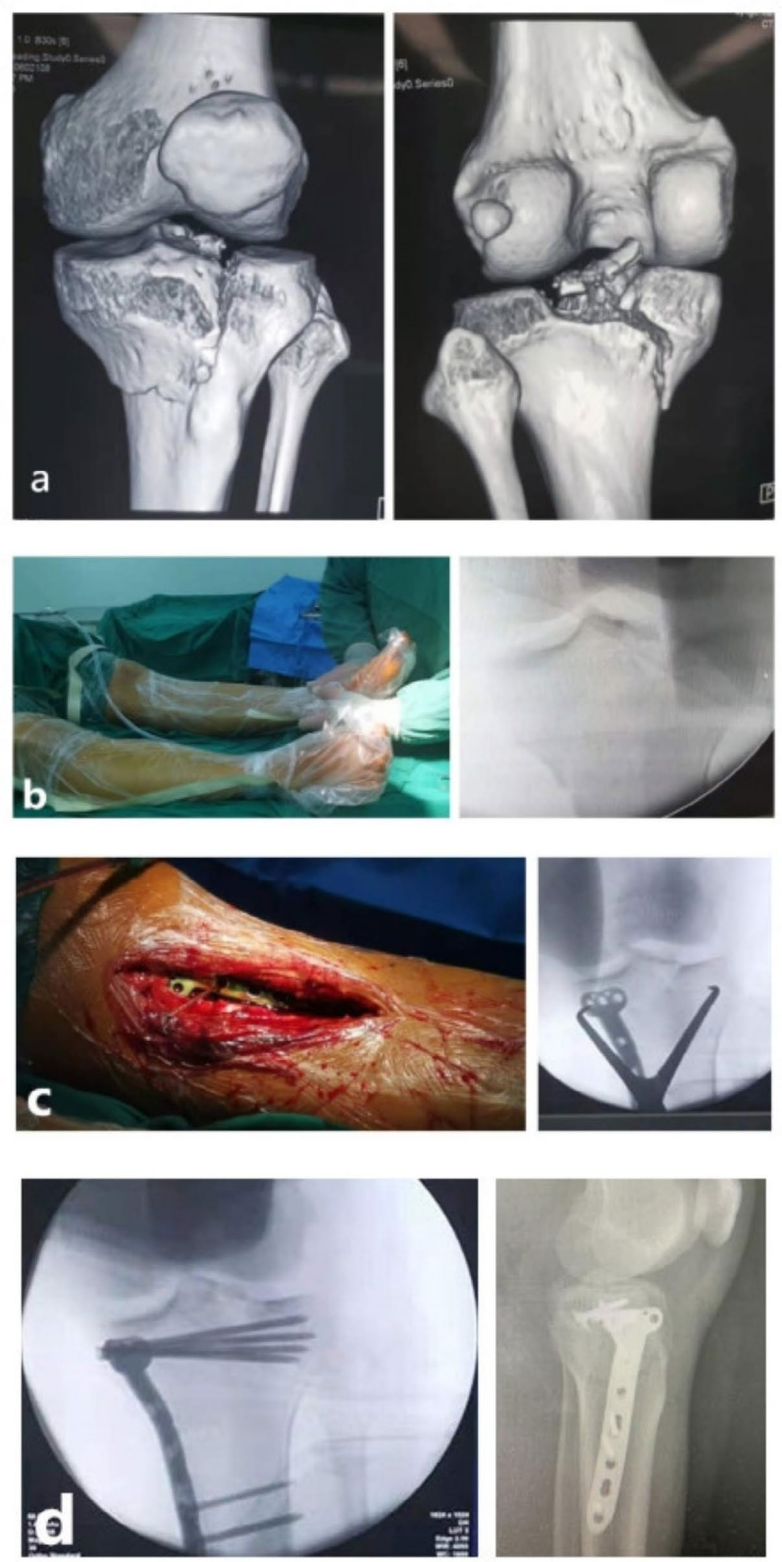
A typical case of a 46-year-old male with Schatzker IV tibial plateau fractures (extension-valgus type). (**a**) The features of this type were shown in CT images. (**b**) For the extension-valgus type, the knee should be placed into extension and perform traction for the affected limb. Intraoperative fluoroscopy showed that the dislocation was nearly corrected. (**c**) After anatomic reduction and temporary fixation, the steel plate was placed upon MFA. Then the patient was turned from a supine position to a prone position and a posterior median incision was made. The posterior intercondylar spine fracture was fixed with screws. (**d**) The anteroposterior and lateral plain radiographs after the operation were shown

A medial approach was performed in both types of injuries (“extension-varus” and “extension-valgus”). Considering the location of the MCZ of extension-varus in medial quadrant, bone graft and plate fixation were performed after the restoration of the medial plateau surface and the reduction of medial condylar fracture. The optimal position of the steel plate was on the MFA. MFA caused by the main deforming force represents the main direction of displacement tendency. Therefore, the reduction of MFA is crucial in the surgical sequence. As for the injuries of *extension-valgus*, the reduction and internal fixation of medial condylar fracture was the first step. Given the location of the MCZ of extension-valgus in the lateral quadrant, fenestration bone grafting under the lateral articular surface combined with internal fixation was performed after the reduction of the lateral articular surface (Figs. 4 and 5).

#### Flexion-varus-internal rotation and flexion-varus-external rotation

**Fig. 6:**
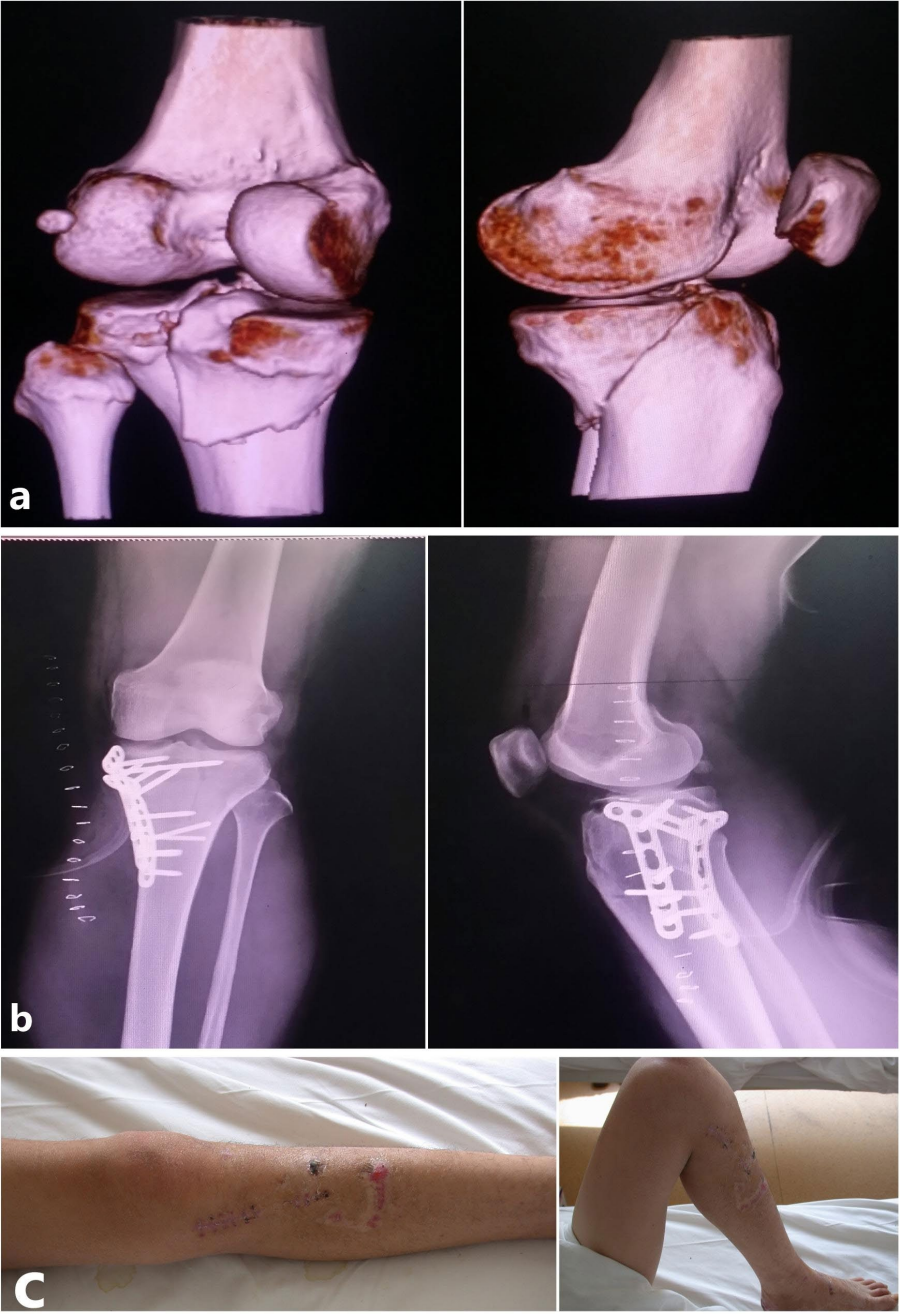
A typical case of a 22-year-old female with Schatzker IV tibial plateau fractures (flexion-varus-internal rotation type). (**a**) The features of this type were shown in CT images. (**b**) After the reduction of MFA, a plate should be placed upon MFA to fix the posteromedial fragment. Another plate was needed to strengthen the fixation of the medial condyle fracture. (**c**) 2 weeks postoperatively, the function of the knee was excellent

**Fig. 7:**
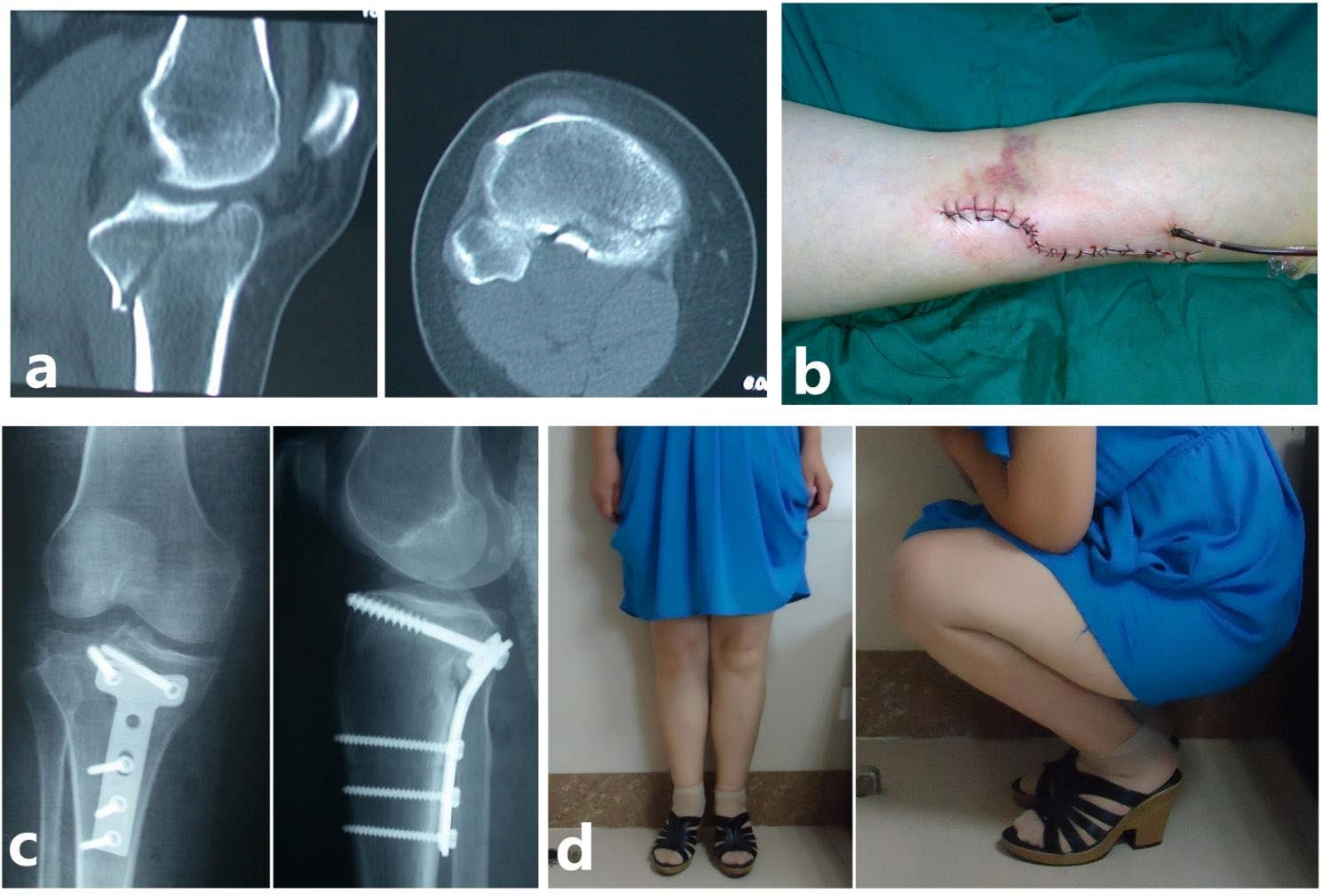
A typical case of a 44-year-old female with Schatzker IV tibial plateau fractures (flexion-varus-external rotation type). (**a**) The features of this type were shown in CT images. (**b**) Carlson Approach was needed for the reduction of MFA. (**c**) After the reduction of MFA, a plate should be placed upon MFA to fix the posteromedial fragment. (**d**) 1 year postoperatively, the function of the knee was excellent

The surgical sequences of the two types of injury were similar. In short, firstly, the knee was placed in an extended position. An S-shape curvilinear incision (Carlson Approach) [12] was needed for the reduction of MFA. The steel plate should be placed upon MFA to fix the posteromedial fragment (Figs. 6 and 7).

#### Flexion-valgus-internal rotation

**Fig. 8:**
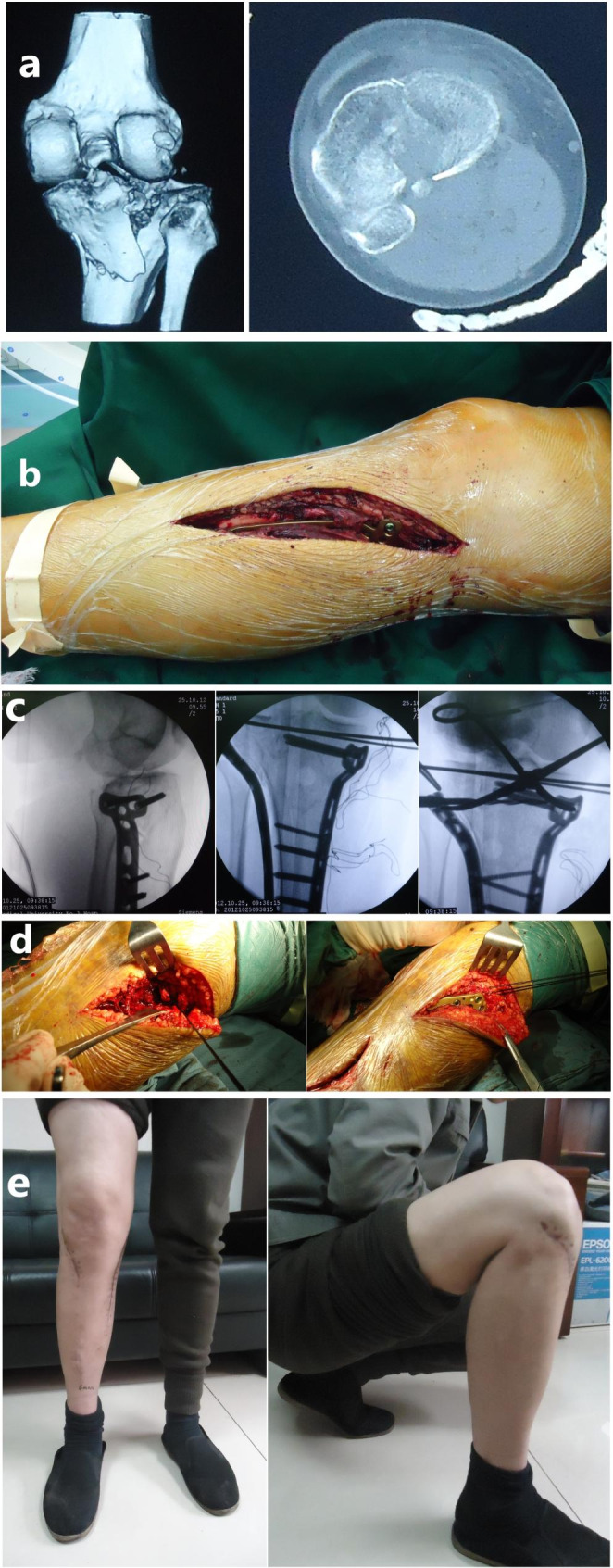
A typical case of a 39-year-old male with Schatzker IV tibial plateau fractures (flexion-valgus-internal rotation type). (**a**) The features of this type were shown in CT images. (**b**) Medial approach was needed for the reduction of MFA. (**c**-**d**) A posteromedial incision was made to expose the posteromedial fracture and MFA in a supine position. After the reduction, the MFA was fixed by screws and a plate that was placed upon the MFA. Then a lateral incision was made to expose MCZ located in posterolateral quadrant and to explore the displaced lateral meniscus. The displaced lateral meniscus and the lateral joint capsule were sutured together. Bone grafting combined with internal fixation was performed after the reduction of the lateral articular surface. (**e**) Postoperatively, the function of the knee was excellent

Firstly, a straight incision was made along the medial tibial ridge to expose the posteromedial fracture and MFA in a supine position. After the correction of joint dislocation and the reduction of posteromedial fracture, a plate was fixed upon MFA. Next, a lateral incision was made to expose MCZ in the posterolateral quadrant and to explore the lateral meniscus. It should be repaired if combined with meniscus injuries. Bone grafting combined with internal fixation was performed after the reduction of the lateral articular surface. If accompanied by avulsion fracture of intercondylar ridge, it should be reduced and fixed at the same time (Fig. 8).

#### Flexion-valgus-external rotation

**Fig. 9:**
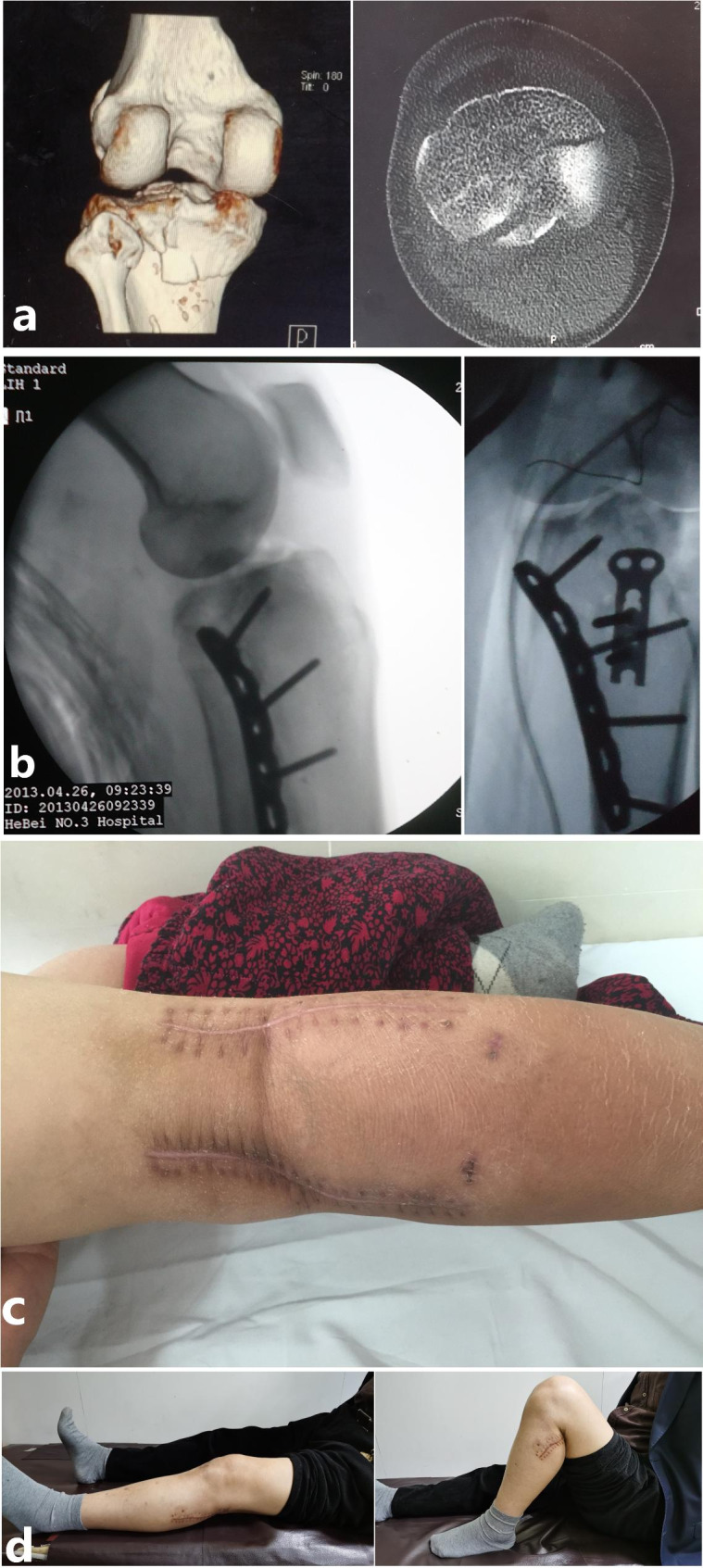
A typical case of a 56-year-old female with Schatzker IV tibial plateau fractures (flexion-valgus-external rotation type). (**a**) The features of this type were shown in CT images. (**b**) The reduction and internal fixation of the medial MFA were performed through posteromedial incision first. Then the lateral MCZ was restored and fixed through the posterolateral approach. (**c**) Bilateral S-shape curvilinear incisions (Carlson Approach) were made for this type of tibial plateau fracture. (**d**) 1 year postoperatively, the function of the knee was excellent

With the patients prone, the bilateral S-shape curvilinear incisions (Carlson Approach) [12] were made for this type of tibial plateau fracture to expose posterior fracture first. Subsequently, the reduction and internal fixation of posterior fracture were performed. As for posterolateral fracture, bone grafting was needed after the reduction to prevent articular surface collapse. Considering the close association of this kind of injury with avulsion fracture of anterior intercondylar spine, patients were then in a supine position for fixation of the avulsion fracture of the anterior intercondylar spine (Fig. 9).

### Postoperative treatment

There is no difference between different types of fractures. From postoperative day 1 to 3, isometric contraction training of the quadriceps femoris muscle was performed to maintain muscle tension. From postoperative day 3 to 7, exercises such as straight leg raising and side leg raising were conducted to strengthen the lower limb muscles. From postoperative weeks 1 to 6, active knee joint flexion exercises were performed. From postoperative weeks 6 to 14, weight-bearing exercises on the affected limb were initiated based on the healing status of the fracture line. Generally, full weight-bearing exercises can be achieved around weeks 12 to 14. Suture removal was typically done at 2 weeks postoperatively, considering the condition of the incision.

### Evaluation of scores the postoperative efficacy

The Knee Society Score (KSS) was used to evaluate the postoperative efficacy of patients [13, 14]. The scoring results are divided into four grades: 80–100 points, excellent; 70–79 points, good; 60–69 points, fair; less than 60 points, poor. Rasmussen’s radiological scoring system evaluates medial tibial plateau fractures based on platform width, tibial plateau articular surface depression, and varus/valgus deformity. The total score ranges from 0 to 18: 18, excellent; 12–17, good; 6–11, fair; 0–5, poor. Two independent experts evaluated the radiographic images and assigned scores based on KSS and Rasmussen’s scoring system. In cases where there was a discrepancy between the two experts’ assessments, we sought the opinion of a third expert (who was considered more authoritative in the field) to provide a final score.

### Statistical analysis

The intra-observer reliability (the same observer in two phases) and the inter-observer reliability (different observers in the same phase) were assessed by the Kappa analysis. The k value ranges from − 1 (meaning complete agreement) to 1 (meaning complete disagreement). Landis and Koch [15, 16] proposed the criteria to introduce the relationship between the k value and the reliability (Table 2). All statistical data in our study were analyzed using the SPSS software (version 25.0). A *P*-value of less than 0.05 indicated a statistically significant difference.

**Table 2:** Landis and Koch grading of reliability

*K* value			Reliability Grading
< 0.00			Poor
0.00–0.20			Slight
0.21–0.40			Fair
0.41–0.60			Moderate
0.61–0.80			Substantial
> 0.80			Excellent

## Results

### Patients

A total of 63 cases (38 men and 25 women) aged 23–76 years (average 45.6 years) were enrolled and followed up for 6–28 months (average 12.8 months). After internal discussion in our team on 3D classification, identical results were achieved as follows: hyperextension-varus-internal rotation in 4 cases, hyperextension-valgus-external rotation in 1 case, extension-varus in 9 cases, extension-valgus in 5 cases, flexion-varus-internal rotation in 6 cases, flexion-varus- external rotation in 3 cases, flexion-valgus-internal rotation in 20 cases, and flexion-valgus-external rotation in 15 cases (Table 3).

**Table 3:** The evaluation of the postoperative efficacy

Classification		KSS							Complication		
		Excellent	Good		Fair		Poor				
H-VR-i H-VL-e E-VR E-VL F-VR-i F-VR-e F-VL-i	4 1 9 5 6 3 20	3 1 9 4 6 2 17		1							
			1								
									1 wound healing delay		
			1								
			2		1				1 wound healing delay;1 incomplete reduction		
F-VL-e	15	11	3		1				1 posteromedial collapse		
Total	63	53	8		2						

Abbreviations of the classification: H = hyperextension; E = extension; F = flexion; VR = varus; VL = valgus; i = internal; e = external

### Reliability of 3D-classification

The reliability of 3D classification was assessed. Both the intra-observer (the mean k = 0.897, CI 0.806–0.971) and inter-observer (the mean k = 0.883, CI 0.786–0.961) reliability of 3D-classification showed excellent agreement according to Landis and Koch. The statistical results are listed in Tables 4 and 5.

**Table 4:** Intraobserver reliability of 3D-Classification

	*K* value	95% CI	
Observer I	0.901	0.808	0.980
Observer II	0.921	0.843	0.980
Observer III	0.862	0.761	0.942
Observer IV	0.902	0.812	0.980
Mean	0.897	0.806	0.971

**Table 5:** Interobeserver reliability of 3D-Classification

Observer	*K* value	95% CI	
I- II	0.921	0.828	0.981
I- III	0.882	0.777	0.961
I- IV	0.843	0.736	0.940
II- III	0.921	0.841	0.981
II- IV	0.882	0.790	0.961
III- IV	0.851	0.745	0.941
Mean	0.883	0.786	0.961

### Evaluation of the postoperative efficacy

According to the grade standard of the KSS system, 53 cases were excellent, 8 cases were good, and the other 2 cases were fair results with relevant complications. Among the 2 cases, 1 case was classified as flexion-valgus-external rotation presenting a loss of reduction with slight posteromedial collapse; another 1 case was classified as flexion-valgus-internal rotation presenting incomplete reduction. Besides, there were 2 cases (1 flexion-varus-internal rotation and 1 flexion-valgus-internal rotation) showing delayed wound healing due to diabetes, which exerted no negative effect on the knee function.

All the included patients were followed up for 14–20 months (average 12.8 months). Regarding Rasmussen’s radiological scoring system, there were no significant differences in the scoring values between postoperative day 1, month 2, month 3, month 6, and month 12 (*p* > 0.05) (Table 6). At the 12-month follow-up, the Rasmussen radiographic scores were as follows: 23 patients were rated as excellent, 38 patients as good, and 2 patients as fair, with an excellent/good rate of 96.8%. In terms of KSS, significant differences in the scoring values were observed between scores at postoperative month 3, month 6, and month 12 (*p* < 0.05). However, the KSS scores at postoperative month 3 and month 6 showed no significant difference (*p* > 0.05), while the KSS scores at postoperative month 12 were significantly higher than those at postoperative month 6 and month 3 (*p* < 0.05). The latest follow-up KSS scores were as follows: 50 patients were rated as excellent, 11 patients as good, and 2 patients as fair, with an excellent/good rate of 96.8%.

**Table 6:** Rasmussen and KSS scores at different periods of postoperative follow-up

Postoperative score	Postoperative follow-up period					*P*-value
	1 day	1 month	3 months	6 months	12 months	
Rasmussen	15.6 ± 2.3	15.8 ± 2.2	15.4 ± 2.1	15.5 ± 2.2	15.7 ± 2.4	>0.05
KSS	N	N	79.8 ± 7.8	82.4 ± 7.2	85.6 ± 6.6	0.013

Noticeably, 2 cases were rated as fair in both postoperative scores (Rasmussen radiographic scores and KSS scores). One case was determined as medial tibial plateau fracture of the flexion-varus-external rotation type, with mild depression of the posterior medial tibial plateau fracture fragment at 12 months postoperatively. The other case had medial tibial plateau fracture of the flexion-varus-internal rotation type, with incomplete reduction and failure to restore tibial plateau width on postoperative imaging.

Additionally, there were 2 patients (1 patient of flexion-valgus-internal rotation type and 1 patient of flexion-varus-internal rotation type) experiencing delayed wound healing complications due to comorbid diabetes. However, these complications did not ultimately impact the postoperative recovery of the knee joint function.

## Discussion

Previously, limited attention has been paid to the injury mechanism of tibial plateau fractures [17]. Although tibial plateau fracture treatment or classification involving the injury mechanism has been discussed, relevant contents were oversimplified [18–21]. In a prior study, Zhang et al. have detailedly discussed the injury mechanism of tibial plateau fractures based on the three-column classification [22]. However, the rotation was not taken into account in this study and the injury mechanism was not completely reflected. As reported by Wang et al., the injury mechanism of Schatzker IV tibial plateau fractures is completely analyzed by simulating knee postures when a fracture occurs [11]. Hence, based on the injury patterns summarized by Wang, this study proposed the 3D classification for Schatzker IV tibial plateau fractures. According to the 3D classification, there were 8 types of injury: hyperextension-varus-internal rotation, hyperextension-valgus-external rotation, extension-varus, extension-valgus, flexion-varus-internal rotation, flexion-varus-external rotation, flexion-valgus-internal rotation, and flexion-valgus-external rotation.

It’s well-estabilished that a popularized classification should be easy to understand and remember. Therefore, the reliability of 3D classification was subsequently assessed in this study. It was found that both the intra-observer and inter-observer reliability of 3D classification showed excellent agreement according to Landis and Koch (*p*<0.01). Each point is easily identified in CT images, with a clear task division for classifying. Taken together, the reliability of the 3D classification exhibited excellent results.

An effective classification should be helpful for treatment [23, 24]. The improper treatment can easily cause complications such as osteoarthritis [25]. Our new 3D classification could determine surgical sequences to some extent in treating Schatzker type IV tibial plateau fractures. The 3D classification-derived surgical sequences for Schatzker type IV tibial plateau fractures were also proposed. All the enrolled patients were treated according to the surgical sequences in our study. According to KSS results, the majority of patients (61 of 63) were rated as excellent/good, and only 2 patients were considered as fair results with relevant complications. No one showed poor results in our study. Among the 2 fair results, 1 case (flexion-valgus-external rotation) presented a loss of reduction with slight posteromedial collapse and the other 1 case (flexion-valgus-internal rotation) presented incomplete reduction. After morphological analysis, flexion-valgus-XX was considered to produce more complex injuries and should be taken seriously.

As evidenced by the results of the evolution of postoperative efficacy, the new surgical sequences were superior in treating Schatzker type IV tibial plateau fractures, which can be attributed to their close association with surgical treatment and injury mechanism. With the definition of “hyperextension”, “extension”, or “flexion”, the first step (the correction of dislocation) would be achieved accurately through the converse injury mechanism.

MFA is also crucial in the second step (the most critical step). MFA caused by the deforming force can to some extent represent the main direction of displacement tendency. After the reduction, the optimal position of the steel plate is upon the MFA for deformity correction. The whole sequence runs based on the injury mechanism. In our study, among 4 cases of *hyperextension-varus-internal* injury, 2 cases were associated with avulsion fracture of fibular capitulum, and 2 cases were associated with posterolateral complex injury. Among 20 cases of *flexion-valgus-internal rotation* injury, 13 cases were complicated with lateral meniscus injury (7 cases with partial dissociation of lateral meniscus, 4 cases with meniscus stuck between fracture spaces, and 2 cases with bucket handle tear). The lateral meniscus injuries were identified by preoperative MRI or diagnosed during operation. Among 15 cases of *flexion-valgus-external rotation* injury, 13 cases were complicated with ACL avulsions that were properly treated. Taken together, it is of great significance to better understand the classification for avoiding combined injuries and improving the treatment effect of Schatzker IV tibial plateau fracture.

The present study highlighted 3 identified points: MFA, MCZ, and MOFL. All these 3 points were easy to recognize and understand for classification and treatment. However, there also exist some limitations in our study. Firstly, the sample size of our study was relatively small (such as 4 cases of hyperextension-varus-internal rotation and 1 case of hyperextension-valgus-external rotation), which limits the generalizability. Secondly, the lack of a control group may cause potential bias in evaluating the postoperative efficacy. Thirdly, the related soft tissue injuries were not analyzed and evaluated. Finally, describing injuries based on force direction may hinder the practicality of surgeons, as it requires analyzing forces, relating them to morphology, and then planning the surgical approach; this process appears overly complex, especially considering that many surgeons rely on CT scans to guide their decision-making.

## Conclusion

The newly proposed 3D classification showed high intra-observer and inter-observer reliability in our study, which indicated that the 3D classification is clear and repeatable, making it easy to remember. Furthermore, this classification-derived surgical sequence showed superior postoperative efficacy in our study. Therefore, these derived surgical sequences can help surgeons achieve optimal reduction and stable internal fixation when managing Schatzker type IV tibial plateau fractures. Therefore, the novel 3D classification and its derived surgical sequences merit further popularization and application in future clinical trials.

## Data Availability

Zihao Liu could provide detailed information if someone wants to request the data from this study. (lzhgg0318@126.com)
